# UK national survey on nuclear medicine in-house clinical software: the calm before the storm

**DOI:** 10.1097/MNM.0000000000001917

**Published:** 2024-10-16

**Authors:** Anthony W. Murray, James W. Scuffham, John C. Dickson, Matthew Memmott

**Affiliations:** a Department of Medical Physics and Clinical Engineering, Leeds Teaching Hospitals NHS Trust, Leeds; b Department of Medical Physics, Royal Surrey NHS Foundation Trust, Guildford; c Institute of Nuclear Medicine, University College London Hospitals NHS Foundation Trust, London; d Nuclear Medicine Centre, Manchester University NHS Foundation Trust, Manchester, UK

**Keywords:** in-house, nuclear medicine, software

## Abstract

**Introduction:**

The use of in-house developed software as a medical device (IHD-SaMD) is core to many nuclear medicine (NM) services in the UK, including applications in nonimaging studies and image processing. Expected regulatory changes in 2025 could have significant implications due to a lack of resources and expertise in the implementation and maintenance of software Quality Management Systems (QMS) and associated standards. This survey investigated the national use of IHD-SaMD and the readiness of services to adapt to the upcoming regulatory changes.

**Method:**

An online survey was used to investigate the current national usage of IHD-SaMD. Representatives of 64 UK NM physics services were invited to participate, with 43 responding.

**Results:**

It was found that 98% of respondents use IHD-SaMD clinically. About 65% use IHD-SaMD that respondents felt was under-supported (e.g. legacy software). Approximately 60% of respondents use or support two or more pieces of IHD-SaMD. Around 66% of respondents use a QMS in their department, with about 48% using a software-specific QMS. Most respondents indicate understaffing, particularly with regard to IT/software skillsets. Almost all respondents indicate without an increase in the preparedness and understanding of the requirements, all dependent clinical services would be severely impacted or indeed stopped.

**Conclusion:**

This national survey shows that pending regulatory changes could significantly impact NM services, up to and including stopping clinical services. Additional resources would be required to support in-house software management under an appropriate QMS or move to European conformity marking (CE)-marked software where available. This must be urgently considered and addressed by all NM stakeholders.

## Introduction

In-house developed clinical software is a core part of UK nuclear medicine (NM) services throughout the UK, such as widely performed glomerular filtration rate (GFR)/bile acid malabsorption calculations, patient dosimetry calculations, and quantitative image processing (e.g. uptake calculations, lymphoscintigraphy, circulatory system shunting quantification), and so on.

Currently, in-house software is widely developed and used to address specific clinical needs that cannot be addressed by available European conformity marking (CE)-marked commercial software [1,2]. Much of this software was developed under the auspices of the Medical Device Regulations (MDRs) 2002, which put into effect in UK law the European Union (EU) Directives for medical devices and in-vitro medical devices (IVMD). Where this software was produced and used within the same health institution and not placed on the market, it was explicitly stated in the IVMD Directive that the regulations did not apply. This has historically been interpreted as the case for non-IVMD, and hence the use and development of in-house software became widespread in nuclear medicine departments.

In 2017, the EU MDR came into force. This formalized the regulation of in-house developed software. While exempting the developers from the full rigors of conformity assessment, developers were subject to the General Safety and Performance Requirements, among other nuances of the Regulations. The main resource-dependent requirement is that all development must be carried out under an appropriate quality management system or software quality management system. In the UK, the Medicines and Healthcare Products Regulatory Agency has suggested that conformance to International Organization for Standardization standards, such as ISO13485, would be considered appropriate. The MDR also requires management processes around risk and product life-cycle, and hence adherence to other standards such as ISO14791 (Risk Management for Medical Devices) and IEC62304 (Medical Device Software Life-Cycle Processes) could also be seen as appropriate. While many departments have accreditation to some standards, such as ISO9001 [3] (Quality Management) and BS70000 (Medical Physics, Clinical Engineering, and associated scientific services in healthcare), these only partially cover the requirements of the above standards and significant gaps would remain. Due to the withdrawal of the UK from the EU in 2020, these regulations were not put into force in the UK, and as such, the 2002 Regulations continue to apply.

The legal landscape in the UK is expected to soon change [4]. In 2024, legislation is expected to be introduced to strengthen postmarket surveillance ahead of core elements of the new MDR framework being put in place in 2025. Depending on transitional arrangements, individual NM departments in the UK will then face the choice of whether or not to maintain the capability to develop, use, and distribute SaMD, which will need additional staff resources to meet the Quality Management requirements. Without this capability, many departments may be forced to stop using in-house developed software as a medical device. This may simply result in additional capital costs where there are CE-marked alternatives. However, there are many applications in nuclear medicine where CE-marked software does not exist. Most notable among these is the GFR test, which is critical to many oncology and renal clinical pathways nationwide. This raises the question: would departments be compelled by the new legislation to stop performing these tests?

There is a national scientific workforce shortage currently [5] which will hinder attempts to meet the new regulatory requirements. Moreover, the effects of these new requirements on in-house software will not be felt uniformly across all NM centers, as typically larger centers will benefit from existing scientific computing teams with software development and management expertise.

In an attempt to collect data on the concerns around changes to UK regulation of in-house NM software, an online anonymous national survey of UK NM physics services was performed to look at the current national status of NM in-house software usage and their current readiness to comply with the range of possible new regulatory requirements.

## Method

The online survey was undertaken in Nov/Dec 2023 to collect the survey information. The questions (Table 1) were chosen to provide a pragmatic overview of the national status of NM in-house software, balancing question brevity and detail to maximize participation. Sixty-five NM physics services were invited to participate with 41 responding, representing a 63% response rate.

**Table 1:** Results of the national survey on UK nuclear medicine in-house software

Response	Percentage	
Do you develop/use/support/maintain in-house produced clinical software as a medical device?	Yes No	97.5% 2.5%
What clinical services does this software support?	GFR calculations BAM calculations Image processing Dosimetry	95.0% 80.0% 47.5% 25.0%
Do you currently support other employers with in-house produced software?	Yes No	22.5% 77.5%
Do you use in-house developed software that is currently less than ideally supported or unsupported (developed by historic staff members, affected by understaffing, etc)?	Yes No	65.0% 32.5%
How many pieces of in-house produced software do you use/maintain/support?	0 1–2 3–4 5–6 6+	0% 40.0% 17.5% 20.0% 22.5%
Do you have a departmental QMS?	None Unaccredited QMS ISO9001 QSI	10.0% 35.0% 32.5% 20.0%
Do you have a departmental SQMS (local system/ procedures, ISO62304, etc)?	None Unaccredited SQMS ISO62304 Part of QMS	42.5% 22.5% 0% 27.5%
In your opinion, do you currently have adequate staffing levels to deal with the workload for your overall service? (Scale: 1, completely understaffed; 5, understaffed; 10, adequately staffed)	1 2 3 4 5 6 7 8 9 10	7.5% .5% 12.5% 12.5% 25.0% 5.0% 10.0% 5.0% 12.5% 2.5%
In your opinion, do you currently have adequate staffing skillsets (IT/software savvy) to managing in-house clinical software under the pending possible regulations? (Scale: 1, completely understaffed; 5, understaffed; 10, adequate staffed)	1 2 3 4 5 6 7 8 9 10	12.5% 15.0% 17.5% 10.0% 22.5% 7.5% 5.0% 10.0% 0% 0%
Does your wider medical physics department/service have a scientific computing team etc or other teams, that develop in-house software?	Yes No	40.0% 52.5%
Do you receive any support from your local IT department with regards to in-house software development?	Yes No	7.5% 87.5%
What software file management structure/systems if any do you use for software quality management?	None Local IT shared drives Github QPulse iPassport EQMS Gitlab	2.5% 57.5% 5.0% 35.0% 5.0% 2.5% 2.5%
Is the risk to the use of all non-CE-marked in-house clinical software, listed on a local risk register?	Yes No	10.0% 85.0%
Which of your clinical services would be impacted if there was a ban on the use of all non-CE-marked in-house clinical software?	GFR calculations BAM calculations Image processing Dosimetry	95.0% 75.0% 50.0% 20.0%

BAM, bile acid malabsorption; CE, European conformity marking; GFR, glomerular filtration rate; QMS, quality management system; SQMS, software quality management system.

## Results

The survey results are shown in Table 1. Some answers are percentages of the total (a total of 100%) and some are the percentages for individual options in each question with multiple nonexclusive options (a maximum total of 100% for each option).

## Discussion

The results show that the pending changes to the regulation of in-house software will have a significant impact on delivering NM services on a national scale, in whichever form the regulations are adopted due to a national lack of resources and expertise.

For example, chemotherapy services relying on NM GFR measurements are particularly vulnerable, with 95% of participating services dependent on in-house software. To add further context, a previous national GFR software audit in 2013 [6] showed that there were approximately 17 000 UK NM GFR studies per year, with 70% being for oncology purposes. Also, NM bile acid malabsorption services will be significantly impacted with 75% of participating NM services relying on in-house software for this procedure. Thousands of these studies are performed nationally each year in the UK [7].

Approximately 50% of participating services use in-house software for imaging processing purposes, which covers a wide range of NM procedures. Lack of patient-specific dosimetry has been identified as a national issue [8] with barriers including a lack of experienced staff and dosimetry software. This issue will be further compounded by restrictions on in-house software.

The Administration of Radioactive Substances Advisory Committee (ARSAC) requires those applying for an ARSAC therapy license to state the method of dosimetry to be used for each radiopharmaceutical therapeutic agent. This is even more pertinent for new therapy agents such as Lutetium-177 prostate-specific membrane antigen. The inability to perform patient-specific dosimetry without in-house software could hinder the ability of medical staff at centers to acquire therapy ARSAC licenses.

There are also challenges supporting existing in-house software due to deficiencies in staffing resources and expertise, in addition to in-house software support offered to other NM centers. About half of the surveyed NM physics services have an accredited quality management system and some form of a software quality management system.

It is expected that small and larger NM centers will be affected differently due to the varying services delivered, infrastructure, expertise (academic, programming, software management, IT skills, etc.), and resource availability. Adequate workforce planning and resource allocation must be considered. Ideally, this will happen in conjunction with all the key stakeholders.

In conclusion, the pending changes to the regulations of in-house software will have a significant impact on UK NM clinical services. This must be carefully addressed at a national level to avoid a significant impact on patient care.

It is hoped that this national survey, in addition to providing citable evidence on this issue, will contribute to current work on the topic of the pending regulation of in-house NM software [9] and to discussions about how to mitigate the impact of such changes on UK NM services. This survey can be used as a baseline for future surveys looking at this issue.

## Acknowledgements

The authors thanks to all the participants.

### Conflicts of interest

There are no conflicts of interest.
